# Listening to the voices: an exploratory study of the experiences of women diagnosed and living with breast cancer in Uganda

**DOI:** 10.11604/pamj.2013.16.60.2431

**Published:** 2013-10-19

**Authors:** Mubuuke Aloysius Gonzaga

**Affiliations:** 1 Radiology department, School of Medicine, College of Health Sciences, Makerere University, P.O. Box 7072, Kampala, Uganda

**Keywords:** Breast cancer, women, experiences, Uganda

## Abstract

**Introduction:**

Breast cancer is one of the leading causes of cancer deaths amongst Ugandan women. Most women live through challenging and emotional experiences having been diagnosed with breast cancer. The purpose of this study was to explore the lived experiences of women diagnosed and living with breast cancer.

**Methods:**

This was an exploratory qualitative study using a convenience sample (n = 12) of women confirmed with breast cancer and reporting to the Radiology department for imaging. In-depth individual interviews were conducted and findings were summarized into themes, representative of the participants’ lived experiences.

**Results:**

All women in this study initially went through emotional trauma of living with breast cancer. However, with time, they seemed to accept and cope with their situation. Four major themes summarizing their experiences included: Thought of Death, Strength to live, Loss of female identity and sexuality and Coping mechanisms.

**Conclusion:**

This study provided a unique insight of the experiences of women living with breast cancer. By listening to their narratives, one could see emotional pain, anger, anxiety, strength to live and many more. Although women with breast cancer get clinical management, there is need to design holistic palliative healthcare services including counseling to assist then cope with life.

## Introduction

Breast cancer is one of the common cancers amongst Ugandan women in addition to Kaposi's sarcoma and cervical cancer [1, 2]. The incidence of breast cancer in Uganda is 22:100,000 [1] and five-year survival rate stands at 56% [3]. Several studies done have also reported that breast cancer is the most common cancer and leading cause of cancer deaths amongst women globally [4–8].

The experience of living with breast cancer is unique to every woman [9, 10]. Such an experience may be influenced by many other factors like support from spouse and family, cultural and religious beliefs, educational and socio-economic background and many more [11, 12]. Women with breast cancer may not only suffer from physical pain, but also emotional, psychological, spiritual and social pain [13, 14]. The individualized experience of breast cancer starts with the shock of test results that confirm breast cancer presence, all through the unexpected journey of breast cancer treatment up to even death [15]. It has been reported in literature that through this journey starting with diagnosis of breast cancer could be characterized by emotional chaos [14, 16]. Some women lose their femininity and sexuality following mastectomy which may lead to loss of supportive spouses [17].

It has also been reported that women diagnosed with breast cancer can lose self-esteem and struggle to acquire emotional coping mechanisms especially after disfigurement following mastectomy [18, 19]. For young women in reproductive years, the early onset of menopause, fears about careers and relationships all compound emotional stress in the midst of confirmed breast cancer [20]. It has been suggested that psychological adjustment is central in this situation and should be based on having a fighting spirit, control of emotional anger and self pity, and possessing a sense of continued hope [16]. Professional support in this situation is also necessary for such women in order for them to cope with the situation. Health professionals also need to provide more support for such women especially on issues relating to counselling and coping with breast cancer in addition to treatment procedures [21].

Breast cancer diagnosis confronts women with the fact that life may never be the same again. Landmark and Wahl [9] reported that women with breast cancer experience a double reaction involving the uncertainty about possible spread to other body organs and the uncertainty about suffering which could lead to death. Women can also lose self-confidence about their own bodies. Keller [10] reported that 20-30% of women treated for breast cancer failed to adapt to living with the disease. Most studies on breast cancer have focused and emphasized clinical aspects of diagnosis, screening and treatment. Some studies have been done on psychosocial aspects of breast cancer, but most of them have focused on social support, life and body image. Few studies have explored the lived experiences of women themselves. Specifically in Uganda, most literature reports about clinical guidelines, diagnosis and clinical management of breast cancer, but silent on how women experience the situation of living with breast cancer. The purpose of this study therefore was to explore the lived experiences of women diagnosed with breast cancer. Understanding the lived experiences of women diagnosed and living with breast cancer can assist health professionals provide adequate care that meets the needs of such women. Additionally, it can also assist policy makers like ministries of health to design programmes that not only cater for the clinical management, but also emphasizes continued overall holistic care of women diagnosed with breast cancer.

## Methods

The study was conducted within the Radiology department of Mulago National referral and teaching hospital in Uganda. It was an exploratory qualitative study using in-depth individual interviews. Questions asked were open ended and explored women's experiences of living with breast cancer. The questions were first piloted before carrying out the interviews. Convenience purposive sampling was used to select the women who participated in the interviews. The women were selected from among those diagnosed and confirmed with breast cancer and reporting to the Radiology department for imaging.

There were twelve women included in the study that had been diagnosed and confirmed with breast cancer and had reported to the Radiology department for imaging. All women confirmed with breast cancer were eligible to participate in the study regardless of age, occupation, background or any other clinical characteristics. The final number of women to be included in the study was not predetermined, but was arrived at by the principle of data saturation. Data collection was terminated at the 12^th^woman since no new themes were emerging. A quiet place was chosen for the interviews. The responses were tape-recorded and later transcribed by the researcher, each interview transcription taking place immediately after carrying out the interview.

Thematic analysis was used for data analysis. This involved proof-reading raw data and coding it into categories of similar meaning. The categories were related to each other leading to the emergence of themes that were used to report findings. Data analysis commenced immediately after the first interview using an iterative process.

The women provided verbal consent after explaining to them the purpose of this study. No participant was identified by name or opinion and all individual responses were kept confidential by the researcher. Data was securely kept in electronic format and secured by a password. Every participant was told that they were free to refuse participating at any time, even free to terminate the interview without any prejudices against them. They were told that this study would not affect their treatment and care in any way. A nursing assistant helped out in accessing potential participants as a third party. At the end of each interview, a nursing assistant once again counseled each lady about breast cancer as a way of calming down any tensions and memories the interview could have invoked.

Lastly, the study received ethical approval from School of Medicine review board of Makerere University.

## Results

Of the twelve women in this study, 2 were married, 3 were divorced and 7 were not in any relationship. Four of the women lived in town while the remaining eight came from rural communities. All the women had received at least primary education. 4 women were between 30 and 40 years, 6 were between 40 and 50 years and the remaining 2 were between 50 and 60 years of age. The results indicated that living with breast cancer provided different experiences related to physical, psychological, social and existential issues. These experiences were summarized into various themes below:

### Thought of Death

This theme seemed to have dominated in the early stages of being diagnosed with breast cancer as women expressed their concerns about the eminent death. There were emotional reactions evident that seemed to be traumatic to the women as they contemplated dying. All women experienced shock after telling them they had breast cancer and all of them thought about death at that point in time as evidenced in the quotes below;

*“It was such a shock that I saw myself in the grave”. “When I was told that I have breast cancer, I knew all was over for me. I immediately started thinking of how am going to leave my children in this world all alone ”. “The news shocked me because I knew my days were numbered. I immediately thought about updating my will because death was just around the corner for me”. “When the doctor told me that I have cancer, I regarded myself as a moving dead body. The whole world seemed meaningless to me"*. However, the most shocking experience was given by a lady who said that: *"I was shattered?. I have always been close to God, but this was unbelievable to be happening to me? I called people at home to start preparing my grave"*.

### Strength to live

This theme was especially active after overcoming the fear of death due to breast cancer. Most of the energy from women seemed to be channeled towards fighting for life and living on. This power to live was related to aspirations for the future, fear for relationships and fear for losing families. A common thread in this theme was the strength for the women to push on even after being diagnosed with breast cancer as evidenced in the following quotes:

*"I have realized that I need to use my remaining time to be good to my family and accomplish what I needed to achieve in life”. “Although I know my life is limited, I will seek strength from the Lord such that I work for my children till I cannot work anymore”. “At first, I cried every day. But after sometime, I realized that it is not the end of the world and I still had something good to contribute not only to my family, but also to my country. Now am at peace”. “After some time of reflection, I realized that I was not the first to get breast cancer and I will not be the last. This gave me strength to continue doing my work and am now pushing on with it"*.

### Loss of female identity and sexuality

This also dominated the responses as all the women felt that being diagnosed with breast cancer was equivalent to losing their female sexuality. This appeared to be associated with repercussions that were likely to develop in their relationships with partners and in their identity as women as evidenced in the following quotes:

*"I know my breast was going to be cut off. I was not crying much for the loss of the breast, but for the loss of my husband because I knew he could not be with me anymore”. “It is the breasts that make up a woman and if I lose them, am becoming a man”. “I do not think I will ever give birth again because if my breasts are cut off due to cancer, how will I breast feed my babies”. “After re-collecting myself, I told all my close friends and relatives to keep quiet about my situation since my wedding was in three months time. The man would have abandoned me and the wedding if he was told that I had breast cancer"*.

### Coping mechanisms

This also emerged as an important theme from the women. All the women in this study demonstrated a strong will to live on with breast cancer and employed diverse methods of coping with the situation. Common among these were; psycho-social support from families and friends, getting closer to God (religious support), engaging in productive activities and remaining healthy with the cancer treatment. Some of these are evidenced in the quotes below:

*"Ever since I was told that I have breast cancer, am now very close to God and have established a personal relationship with Him. He is now my comfort”. “Members of my church have helped a lot in living with the situation. I now have no more fears”. “I thank my husband, children, relatives and friends who have given me encouragement and support. I now feel healthy and carrying on with my activities”. “I take my medicine as given to me by the doctor. I think this has kept me well and alive. I still have hope that with this medicine and prayers to my God, I will get cured of this cancer"*.

## Discussion

Results from this study indicate that living with breast cancer comes with a lot of psycho-social challenges to women in support of what Land mark and Wahl reported [9]. The initial experience by women of seeing eminent death is most likely due to the shock from learning that they had breast cancer and yet they are aware that cancer has no cure. The women probably considered themselves unhealthy anymore because of the stigma that sometimes comes with having breast cancer. Most likely these women viewed life as no longer taking their planned course, but being dictated by their present condition.

However, after some time, the women appeared to have accepted and adapted to the situation to live with breast cancer. It is most likely that after reflection about their situation, the women realized that they still had a life to live if they positively accept their situation. Therefore, fighting to survive seemed to give the women a new meaning to life, an observation reported in other studies [22–24]. Additionally, women in this study probably viewed health and life as being precious over death. This could explain the reason why women who initially thought about death, later gained strength to move on. The tenacity of death can therefore be viewed as a driving force that supports women to fight on despite the sufferings and initial fears associated with breast cancer.

In this study, it was apparent that women went through a lot of emotions when they were told that they had breast cancer, however as time passed on, these reactions seemed to regress and women gained more confidence. This is probably because of the time lag that allowed women to mediate and reflect upon their situation. This self-reflection could have allowed them to gain more control of their lives and the spirit to continue living. Keller [10] reported that people adapt to emotional changes with time, an observation that was evident in this study. Probably what women went through were normal defence mechanisms that assisted them to overcome anxiety.

However, this time of reflection for women is likely to be more fruitful if they receive emotional and social support from family, relatives, friends and even health professionals. The importance of psycho-social support emerged as a strong motivator for the women to cope. Conversely, if a woman with breast cancer is abandoned by her people, it may trigger a sense of worthlessness, and eventually accelerate her death. This emotional support is particularly needed from spouses, friends and relatives to give the women strength. Unfortunately, like some women expressed, some of these anchors of support disappear on learning that the woman has breast cancer. This therefore stresses the importance of continued support for women living with breast cancer.

The diagnosis of breast cancer in most cases awakens fear, anxiety, worthlessness, anger, doubt and self-pity. However, like this study has shown, it appears like all these are transitory and women eventually develop coping mechanisms to live on [25]. In this study, women showed a variety of coping mechanisms like getting closer to God, interacting with family and friends, actively engaging in productive activities and many more. However, in adapting to these coping mechanisms, the women still need support from all stakeholders to assist them through. This is especially important in situations where femininity and sexuality of the women is threatened. Many women in this study expressed fear in losing their sexuality including loss of spouses. This also calls for participation of men in supporting their spouses when they get breast cancer. Knowledge needs to be disseminated that loss of a breast for example through mastectomy does not relegate a woman to something else, but she retains her femininity and sexuality. Cohen et al [26] of a breast does not take away the social image of a woman and this is what all health professionals should be preaching.

Additionally, women diagnosed and living with breast cancer are still normal and active people and should be supported to engage in economically and socially productive activities. This also applies to positions of employment and relationship situations. This study has demonstrated that women living with breast cancer wish to regain their former patterns of living and should not be isolated by those who are essentially supposed to assist them achieve this goal. Landmark et al [9] advise that living with breast cancer does not only affect the woman involved, but also affects her social networks and therefore such women need all the support from spouses, relatives, family and friends to cope with life.

It should be observed that none of the women mentioned receiving psycho-social support from health care providers. Although women diagnosed with breast cancer receive cancer treatment from health workers, they still need professional support from these health workers like counseling and continued holistic palliative care. There is need for policy makers in ministries of health to design nation- wide health care support interventions for women living with breast cancer as seen in cases of HIV/AIDS.

Although this study has documented some of the lived experiences of women living with breast cancer, there are limitations worth noting. Firstly, the small number of participants in this study may not necessarily portray the experiences of all women living with breast cancer in Uganda. Many more exploratory studies of this nature are needed to supplement this. Secondly, this study did not discriminate between women who had been newly diagnosed with breast cancer and those who had lived with it for some time and probably receiving treatment. These two categories of women probably have differing experiences and more studies are needed to compare experiences of various strata of women diagnosed and living with breast cancer. However, the study provides a basis for many more studies on this subject.

## Conclusion

This study has demonstrated that women go through challenging experiences after being diagnosed with breast cancer starting including even thoughts of death and anxiety. Although they develop coping mechanisms, these women need psycho-social support from friends, family, spouses and relatives to cope. Health workers are particularly encouraged to go beyond cancer treatment, but offer holistic approaches including counseling to such women. The ministries of health are particularly called upon to design palliative care interventions for women living with breast cancer as has been done with HIV/AIDS.

## References

[1] Wabinga HR, Parkin DM, Wabwire-Mangeni F, Namboze S (2000). Trends in Cancer incidence in Kyadondo County, Uganda. Br J Cancer..

[2] Gakwaya A, Galukande M, Luwaga A, Jombwe J, Faulal J, Kiguli-Malwadde E (2008). Breast cancer guidelines for Uganda. African Health Sciences.

[3] Gakwaya A, Kigula-Mugambe JB, Kavuma A, Luwaga A, Fualal J, Jombwe J, Galukande M, Kanyike D (2008). Cancer of the breast: 5 year survival in a tertiary Hospital in Uganda. Br J Cancer..

[4] Dibble SL, Vanoni JM, Miaskowski C (1997). Women′s attitudes toward breast cancer screening procedures: Differences by ethinicity. Womens Health Issues..

[5] Ferro S, Caroli A, Nanni O (1992). A Cross-sectional survey on breast self examination practice, utilization of breast professional examination, mammography and associated factors in Romagna, Italy. Tumori..

[6] Odusanya OO, Tayo OO (2001). Breast cancer knowledge, attitudes and practice among nurses in Lagos, Nigeria. Acta Oncol..

[7] Sadler GR, Dhanjal SK, Shah NB (2001). Asian Indian women: Knowledge, attitudes and behaviour toward breast cancer early detection. Public Health Nurs..

[8] Sadler GR, Ryujin LT, Ko CM (2001). Korean women: Breast cancer knowledge, attitudes and behaviours. BMC Public Health..

[9] Mark BT, Wahl A (2002). Living with newly diagnosed breast cancer: a qualitative study of 10 women with newly diagnosed breast cancer. J Adv Nursing..

[10] Keller M (1998). Psychosocial care of breast cancer patients. Anticancer Research..

[11] Im EO, Lee EO, Park YS (2002). Korean women′s breast cancer experience. Western J. Nursing Research..

[12] Taleghani F, Yekta ZP, Nasarabadi AN (2006). Coping with breast cancer in newly diagnosed Iranian women. J Adv Nursing..

[13] Arman M, Rehnsfeldt A, Lindholm L, Hamrin E (2002). The face of suffering among women with breast cancer-being in a field of force. Cancer Nursing..

[14] Perreault A, Bourbonnais FF (2005). The experience of suffering as lived by women with breast cancer. Int J Palliat Nurs.

[15] Kralik D, Brown M, Koch T (2001). Women's experiences of “being diagnosed" with a long-term illness. J Adv Nurs.

[16] Landmark BT, Strandmark M, Wahl A (2001). Living with newly diagnosed breast cancer-the meaning of existential issues. Cancer Nursing..

[17] Davies M, Sque M (2002). Living on the outside looking in: a theory of living with advanced breast cancer. Int J Palliat Nurs.

[18] Cohen MZ, Kahn DL, Steeves DH (1998). Beyond body image: the experience of breast cancer. Oncol Nurs Forum..

[19] Brown M, Koch T, Webb C (2000). Information needs of women with non-invasive breast cancer. J Clin Nursing..

[20] Knobf M (2002). Carrying on the experience of premature menopause in women with early stage breast cancer. Nursing Research..

[21] Vivar CG, McQueen A (2005). Informational and emotional needs of long-term survivors of breast cancer. J Advanced Nursing..

[22] Coward DD (1998). Facilitation of self-transcendence in breast cancer support group. Oncol Nurs Forum..

[23] Landmark BT, Strandmark M, Wahl AK (2002). Breast cancer and experiences of social support. Scand J Caring Sci..

[24] Kahn DL, Steeves RH (1995). The significance of suffering in cancer care. Semin Oncol Nurs.

[25] Carlsson M, Hamrin E (1994). Psychological and psychosocial aspects of breast cancer and breast cancer treatment: a literature review. Cancer Nurs.

[26] Cohen MZ, Kahn DL, Steeves DH (1998). Beyond body image: the experience of breast cancer. Oncol Nurs Forum.

